# A New Approach for Loading Anticancer Drugs Into Mesenchymal Stem Cell-Derived Exosome Mimetics for Cancer Therapy

**DOI:** 10.3389/fphar.2018.01116

**Published:** 2018-09-26

**Authors:** Senthilkumar Kalimuthu, Prakash Gangadaran, Ramya Lakshmi Rajendran, Liya Zhu, Ji Min Oh, Ho Won Lee, Arunnehru Gopal, Se Hwan Baek, Shin Young Jeong, Sang-Woo Lee, Jaetae Lee, Byeong-Cheol Ahn

**Affiliations:** ^1^Department of Nuclear Medicine, School of Medicine, Kyungpook National University, Daegu, South Korea; ^2^Department of Nuclear Medicine, Kyungpook National University Hospital, Daegu, South Korea

**Keywords:** MSC, exosome mimetic, paclitaxel, breast cancer, MDA-MB-231

## Abstract

Exosomes derived from mesenchymal stem cells (MSCs) have been evaluated for their potential to be used as drug delivery vehicles. Synthetically personalized exosome mimetics (EMs) could be the alternative vesicles for drug delivery. In this study, we aimed to isolate EMs from human MSCs. Cells were mixed with paclitaxel (PTX) and PTX-loaded EMs (PTX-MSC-EMs) were isolated and evaluated for their anticancer effects against breast cancer. EMs were isolated from human bone marrow-derived MSCs. MSCs (4 × 10^6^ cells/mL) were mixed with or without PTX at different concentrations in phosphate-buffered saline (PBS) and serially extruded through 10-, 5-, and 1-μm polycarbonate membrane filters using a mini-extruder. MSCs were centrifuged to remove debris and the supernatant was filtered through a 0.22-μm filter, followed by ultracentrifugation to isolate EMs and drug-loaded EMs. EMs without encapsulated drug (MSC-EMs) and those with encapsulated PTX (PTX-MSC-EMs) were characterized by western blotting, nanoparticle tracking analysis (NTA), and transmission electron microscopy (TEM). The anticancer effects of MSC-EMs and PTX-MSC-EMs were assessed with breast cancer (MDA-MB-231) cells both *in vitro* and *in vivo* using optical imaging. EMs were isolated by the extrusion method and ultracentrifugation. The isolated vesicles were positive for membrane markers (ALIX and CD63) and negative for golgi (GM130) and endoplasmic (calnexin) marker proteins. NTA revealed the size of MSC-EM to be around 149 nm, while TEM confirmed its morphology. PTX-MSC-EMs significantly (*p* < 0.05) decreased the viability of MDA-MB-231 cells *in vitro* at increasing concentrations of EM. The *in vivo* tumor growth was significantly inhibited by PTX-MSC-EMs as compared to control and/or MSC-EMs. Thus, MSC-EMs were successfully isolated using simple procedures and drug-loaded MSC-EMs were shown to be therapeutically efficient for the treatment of breast cancer both *in vitro* and *in vivo*. MSC-EMs may be used as drug delivery vehicles for breast cancers.

## Introduction

Studies have investigated the possible applications of synthetically isolated vesicles from cells, called as exosome-mimetics (EMs), as drug delivery vehicles (Fais et al., 2013; Jang et al., 2013). Liposomes, carbon nanotubes, gold nanoparticles, and dendrimers are some of the most common drug delivery systems (DDSs) that have common disadvantages such as adverse side-effects related to immunogenicity and accumulation in kidneys or the liver (Estanqueiro et al., 2015; Pérez-Herrero and Fernández-Medarde, 2015; Zhu and Liao, 2015; Sajid et al., 2016). In addition, DDSs exhibit reduced *in vivo* circulation time, owing to their premature elimination by the immune system (Pérez-Herrero and Fernández-Medarde, 2015; Szebeni and Storm, 2015). Extracellular vesicles (EVs), including exosomes and microvesicles, are lipid membrane-bound vesicles secreted from all types of cells. EVs comprise a variety of molecules such as proteins, RNAs, and miRNAs that have originated from native cells and these molecules are transferred to other cells (Skog et al., 2008; Gangadaran et al., 2017b; Rajendran et al., 2017). Although EVs may serve as feasible DDSs, they have limitations such as low production yield and complicated procedures (Jang et al., 2013; Gangadaran et al., 2018). EMs display characteristics similar to EVs, such as membrane properties of the parent cells (Roth et al., 2008). In comparison with EVs, EMs offer advantages of low cost and high production yield (Jang et al., 2013; Jo et al., 2014).

Paclitaxel (PTX) is one of the effective antitumor agents against advanced and early-stage cancers, including breast cancers (Rowinsky and Donehower, 1995). However, PTX therapy is known to damage healthy cells (Schiff et al., 2009); hence, specific delivery of PTX to cancerous tissues is desirable to reduce the adverse effects to normal cells. Breast cancer is the most common cancer and a major cause of cancer-related death in females worldwide (Zheng et al., 2014). The antitumor properties of PTX against breast cancer are attributed to microtubule polymerization and mitotic arrest of cells at the metaphase/anaphase. However, this beneficial effect is often associated with severe side-effects such as cardiotoxicity, myelosuppression, and neurotoxicity (Marupudi et al., 2007; Gornstein and Schwarz, 2014). Therefore, drug delivery strategies targeted specifically to cancers could improve the therapeutic outcome in terms of patient survival. Therapies that reduce systemic toxicity would provide better treatment efficacy in breast cancer patients. Encapsulation of drugs in a tumor-targeting material was successfully achieved by Jang et al., and doxorubicin-loaded exosomes and nanovesicles were used as efficient delivery systems for cytotoxic drugs (Jang et al., 2013).

Extracellular vesicles loaded with chemotherapeutics may offer interesting drug delivery applications, as targeted delivery could reduce the adverse effects of chemotherapy. The uptake of chemotherapeutic-loaded EVs by the cancerous cells may result in their death and decrease the drug-mediated adverse effects (Tang et al., 2012). However, several mammalian normal cells release low concentrations of vesicles (Lakhal and Wood, 2011; van Dommelen et al., 2012). In this direction, we isolated exosome mimetics (EMs) from mesenchymal stem cells (MSCs) mixed with PTX by extrusion and tested their potentials as nanodrug delivery systems. The main aim of this study was to develop PTX-loaded EMs as effective therapeutic agents for treating breast cancer.

## Materials and Methods

### Culture and Characterization of MSCs

We purchased human bone marrow derived mesenchymal stem cells from the American Type Culture Collection (ATCC). The cells were grown in Dulbecco’s modified Eagle’s medium-F12 (DMEM-F12) supplemented with 10% fetal bovine serum (FBS) and MSC growth supplement. Cells from passage number 4 to 8 were used for experiments. The expression of stem cell markers, CD44 and CD45, was analyzed by flow cytometry (BD Accuri^TM^ C6; BD Biosciences, San Jose, CA, United States).

### Cell Culture of MDA-MB-231, MCF-7, and MCF-10A

The triple-negative breast cancer cell line MDA-MB-231 and another breast cancer cell line MCF-7 was grown in DMEM-high medium supplemented with 10% FBS and 1% antibiotic-antimycotic solution (Gibco). The normal breast cell line MCF-10A was kind gift from Professor. Byungheon Lee (Department of Biochemistry and Cell Biology, School Medicine, Kyungpook National University). MCF-10A cells were grown in the mammary epithelial base media (MEBM) with growth supplements bovine pituitary extract (BPE), hEGF, hydrocortisone, insulin, with antibiotic-antimycotic solution at 37°C in a humidified atmosphere of 5% CO_2_ incubator.

### Transduction of Enhanced Firefly Luciferase (Effluc) Gene Into MDA-MB-231 Cells

The effluc gene was transduced in MDA-MB-231 cells by effluc-expressing retroviral particles isolated from Phoenix A-effluc cells. The transduced MDA-MB-231/effluc cells were sorted with magnetic bead sorting protocol using CD90.1 (thy1) antibody MicroBeads (MACS, Miltenyi Biotec, Germany) to obtain effluc-positive cells. The effluc activity was confirmed with the addition of the substrate D-luciferin and using IVIS Lumina III imaging system.

### Isolation of Exosomes

Exosomes were cultured in exosomes-depleted FBS (produced by centrifugation at 120,000g for 18 h at 4°C), then exosomes were isolated using differential centrifugation. Briefly, cellular debris were removed from cell culture media by sequentially centrifuged. Then cell free supernatant was then filtered through a 0.22-μm syringe filter, before ultracentrifugation at 100,000g for 60 min. Pellets were washed with phosphate-buffered saline (PBS) before a second 60 min ultracentrifugation at 100,000g. All ultracentrifugation steps were performed using an Optima^TM^ L-100 XP ultracentrifuge with Ultra-Clear tubes (Beckman Coulter). All centrifugations were performed at 4°C.

### Preparation of EM and PTX-Loaded EM

Paclitaxel was purchased from Selleckchem (United States). The stock solution was prepared in absolute methanol at a concentration of 5 mg/mL. EM was prepared by slight modifications in the previously reported protocol (Jang et al., 2013). Cells were resuspended at a density of 4 × 10^6^ cells/mL in phosphate-buffered saline (PBS) and treated with different concentrations of PTX (25, 50, and 100 μg) or without PTX and extruded using polycarbonate membrane filters with varying pore sizes (10, 5, and 1 μm) in a mini-extruder (Avanti Polar Lipids). After extrusion, the materials were centrifuged to remove debris and filtered through 0.22-μm filters, followed by ultracentrifugation for 1 h at 100,000 × *g* at 4°C. EMs and PTX-loaded EMs were obtained after washing with PBS following ultracentrifugation.

### Size Measurement by Nanoparticle Tracking Analysis (NTA)

Both MSC-EMs and PTX-MSC-EMs were diluted to 1 μL/mL in distilled water for size measurement using the NanoSight LM10 instrument, and 1 mL of MSC-EM or PTX-MSC-EM was introduced into the viewing chamber by a disposable syringe. On black background, individual EM particles appeared as point-scatters moving under Brownian motion that were quantified by the NTA software. The software automatically tracked and measured the size of MSC-EMs and PTX-MSC-EMs. The NTA frequency size distribution graph was plotted.

### Quantification of PTX With UV-VIS Spectrophotometer

For standard curve generation, PTX was diluted from 0 to 10 μg/mL concentrations and the presence of PTX in MSC-EMs was demonstrated by a UV-VIS spectrophotometer. MSC-EV and PTX-MSC-EV samples were mixed with radioimmunoprecipitation assay (RIPA) buffer and analyzed by measuring UV absorbance at 230 nm wavelength. The absorbance was calculated as OD of PTX-MSC-EM – OD of MSC-EM these value was plotted to standard curve of the PTX (μg/mL). The proteins in EM samples were quantified by the bicinchoninic acid method (Pierce^TM^ BCA Protein Assay Kit). The PTX present in the EM was calculated as follows: Amount of PTX present in the EM (μL) / microgram of EM protein. PTX concentration in EM was expressed as nanogram of PTX per microgram of protein.

### Transmission Electron Microscopy (TEM) Analysis

For TEM analysis, EM samples were fixed with 2.5% glutaraldehyde and 10 μL of EM suspension was placed on parafilm. A formvar-coated copper grid was softly placed on the top of the drop for 20 min in a humidified chamber. Grids were washed with PBS (pH 7.4) and EM was stained with 2% uranyl acetate for 10 min. The grid was washed three to four times with PBS, air dried, and observed under a transmission electron microscope (Hitachi, Tokyo, Japan) operated at 100 kV.

### Uptake of EMs Into MDA-MB-231 Cells

To confirm the encapsulation of PTX in EMs, 4 × 10^6^ MSCs were mixed with 1 μM of OregonGreen-labeled PTX (OG-PTX) (Invitrogen) and extrusion was performed. A total of 5 μL/mL of EM was labeled with DiD, a lipophilic dye, for 30 min and washed twice with PBS. The samples were subjected to ultracentrifugation and 10 μg of DiD-labeled EMs loaded with OG-PTX were incubated with MDA-MB-231 cells for 4 h in a four-well chambered slide. After 4 h treatment, cells were washed twice with PBS and fixed with 4% paraformaldehyde for 10 min. The cells were washed twice with PBS and subjected to DAPI (4′,6-diamidino-2-phenylindole) staining using Vecta mounting medium containing DAPI (Vector Laboratories, Burlingame, CA, United States). The cellular uptake of OG-PTX or OG-PTX-MSC-EM was observed under confocal laser microscopy (Zeiss, LSM, Airyscan, Germany).

### Fluc Activity for Cell Viability

MDA-MB-231/effluc (5 × 10^3^ cells) were plated in a 96-well plate and treated with different concentrations of MSC-EMs and PAC-MSC-EMs (2.5, 5, and 10 μg). After 24 and 48 h, the Fluc activity of MDA-MB-231 cells was measured by IVIS Lumina III imaging system. The measured activity was quantified by drawing region of interest (ROI) over the well and then calculating the relative Fluc activity.

### Cell Viability Analysis

MDA-MB-231/effluc cells treated MSC-EM and PTX-MSC-EM (2.5, 5, and 10 μg) for 24 and 48 h. In addition, another breast cancer MCF-7 cells were treated with MSC-EM and normal MCF-10A cells treated with PTX-MSC-EM (2.5, 5, and 10 μg), after treatment period the medium was removed and cells were treated with 0.5 mg/mL of 3-(4,5-dimethylthiazol-2-yl)-2,5-diphenyltetrazolium bromide (MTT) for 90 min. The insoluble formazan crystals formed in the cells were dissolved with dimethyl sulfoxide (DMSO) and the developed color intensity was measured at 550 nm in a microplate plate reader (Bio-Rad). The relative cell viability was calculated and plotted on a graph.

### Tumor Model and *in vivo* Antitumor Effects

All experiments and animal models were approved by the Institutional Animal Care and Use Committee at KNU, Daegu, Republic of Korea (approval number: KNU-2012-43). Nude BALB/c mice (6-weeks old) were purchased from Hamamatsu, Shizuoka, Japan, and subcutaneously engrafted with 5 × 10^6^ breast cancer cells (MDA-MB-231/effluc) in the right flank with Matrigel (1:3 dilution). The tumor growth was confirmed with the Fluc activity of MDA-MB-231 tumor. Mice were randomly divided based on the Fluc activity at day 1 into three groups, namely, control (PBS), MSC-EM, and PTX-MSC-EM. MSC-EM (50 μg) or PTX-MSC-EM (50 μg) was injected by an intratumoral injection at day 1 and day 5. During the treatment period, Fluc activity was intermittently monitored. At day 8, the mice were sacrificed, and the tumors were excised and weighed.

### Statistical Analysis

The data were analyzed and expressed as mean ± standard deviation (SD). Significance between groups were analyzed by Student’s *t*-test and the graphs were plotted in the GraphPad Prism5 software version 5.01 (GraphPad Software, Inc., United States). A value of *p <* 0.05 was considered significant.

## Results

### Characterization of MSCs

The expression of phenotypic markers in MSCs was confirmed using flow cytometry. MSCs showed more than 95% expression of CD44 but were negative for CD45 (**Supplementary Figure S1**).

### Fluc Activity of MDA-MB-231/Effluc Cells

The Fluc activity of MDA-MB-231/effluc cells was measured with IVIS Lumina III imaging system (Perkin-Elmer). The Fluc activity of MDA-MB-213/effluc cells increased with an increase in the cell number. The quantitatively measured data confirmed the serial increase in Fluc activity (**Supplementary Figure S2**; *R*^2^ = 0.91).

### Characterization of EMs

**FIGURE 1:**
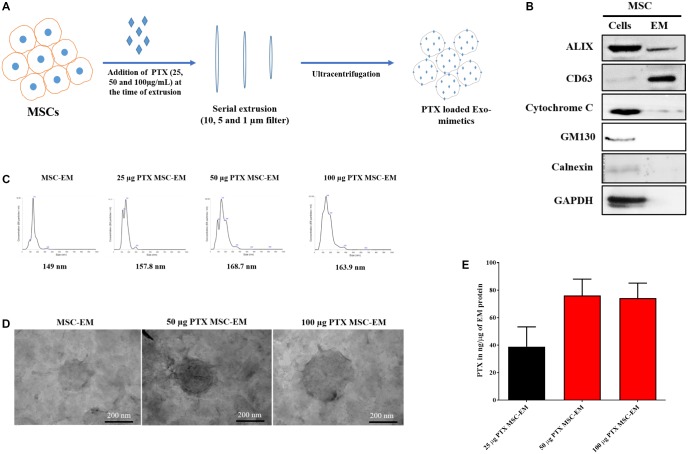
Characterization of EMs isolated from MSCs. **(A)** Schematic diagram of the process of generation of EMs from MSCs. **(B)** Marker protein analysis by western blotting. ALIX and CD63 were positive extracellular vesicle marker proteins. The negative markers, calnexin (endoplasmic reticulum marker) and GM-130 (golgi marker), were undetected in MSC-EMs. **(C)** Analysis of particle size of MSC-EMs and PTX-MSC-EMs by NTA. The particle size of MSC-EMs was around 149 nm, whereas that of drug-loaded EMs was slightly higher. **(D)** Analysis of MSC-EMs and PTX-MSC-EMs by TEM (Scale bars, 200 nm). **(E)** Quantitative measurement of PTX loaded into EMs when 25, 50, or 100 μg/mL of PTX was added to MSC suspension solution during extrusion, as measured by UV spectrometry. Values are expressed as the mean ± standard deviation (SD) of three experiments.

**FIGURE 2:**
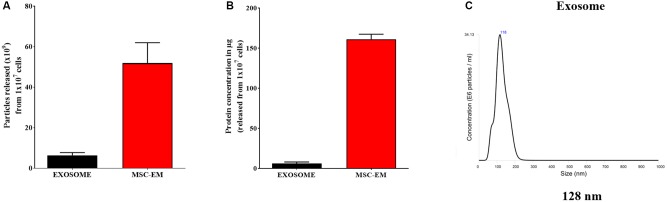
Protein concentration and particle size analysis. **(A)** Protein concentration of MSC-EMs and exosomes isolated from the same number of MSCs. **(B)** Particle number quantification analysis by NTA for exosomes and EMs. **(C)** Exosome size analysis by NTA.

We collected MSCs and extruded through polycarbonate membranes of 10-, 5-, and 1-μm pore sizes to obtain MSC-EMs (**Figure 1A**). These MSC-EMs were analyzed for the expression of exosomal markers with western blotting. ALIX, a vesicle trafficking-related protein present in cells, and CD63 (tetraspanin protein) that is often recognized as a vesicular protein were present in EMs. On the other hand, cytochrome c (mitochondrial marker), GM130 (golgi apparatus marker), and calnexin (endoplasmic reticulum marker) were confirmed in cell lysates but not in EMs (**Figure 1B**). Furthermore, we analyzed the size and shape of purified EMs by NTA and TEM. NTA revealed the average size of EMs to be around 149 nm (**Figure 1C**). TEM analysis highlighted the morphology of EMs to be spherical (**Figure 1D**). The concentration of proteins isolated from MSC-EMs (161 μg) was higher than those isolated from exosomes (6 μg) obtained from the same number of MSCs (1 × 10^7^ cells). The number of particles released from MSC-EMs (51.8 × 10^9^ particles) was higher than that of exosomes (6 × 10^9^ particles) obtained from the same number of MSCs (**Figures 2A,B**). We confirmed the size of the exosomes to be around 128 nm (**Figure 2C**). These results suggest that MSC-derived EMs were similar to MSC-derived exosomes in terms of size and morphology.

### Loading Efficiency of PTX in EM

To load PTX in EM, we first collected MSCs and incubated these with different concentrations of PTX during extrusion. The size, shape, and encapsulation efficiency of PTX-MSC-EM was evaluated. In comparison with the size of MSC-EM (149 nm), the size of EM gradually increased to 157.8, 168.7, and 163.9 nm after PTX loading at 25, 50, and 100 μg/mL concentrations, respectively (**Figure 1C**). Furthermore, PTX loading efficiencies in MSC-EM increased with an increase in the concentrations of PTX from 25 to 100 μg/mL; however, no further increase in the loading efficiency was observed at concentrations of 100 μg/mL, suggesting that the best loading concentration was 50 μg/mL. The loading efficiency was 38.9, 76.1, and 74.22 ng/μg of EM proteins with 25, 50, and 100 μg/mL concentrations of PTX, respectively (**Figure 1E**).

### Cellular Uptake of PTX-MSC-EMs

**FIGURE 3:**
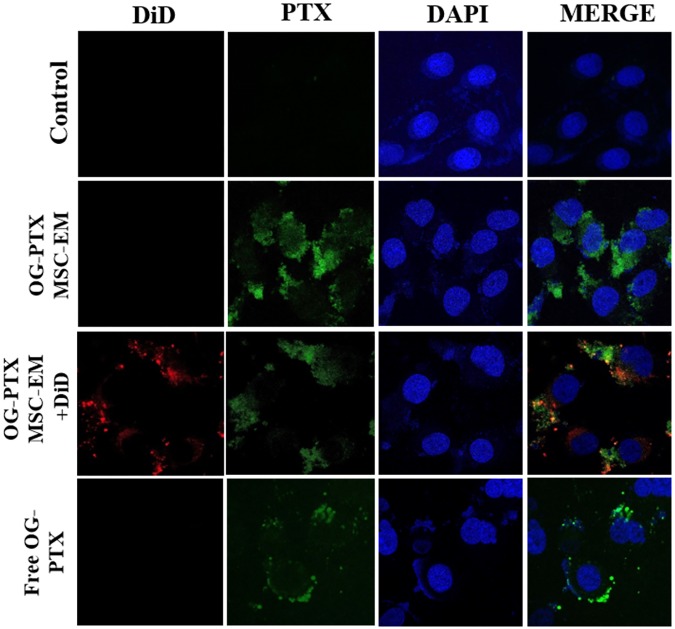
Uptake of PTX by confocal microscopy. MSC-EMs and OG-PTX-MSC-EMs were isolated with extrusion and labeled with DiD. These were incubated with MDA-MB-231 cells. After 4 h, the cells were analyzed by confocal microscopy. Scale bar, 20 μm.

After 4 h incubation with MSC-EMs and PTX-MSC-EMs, MDA-MB-231 cells showed uptake of EMs. MDA-MB-231 cells incubated with DiD-labeled EMs loaded with OG-PTX revealed the colocalization of DiD and OG in the cells, indicating that PTX-MSC-EMs were successfully internalized by MDA-MB-231 cells. MDA-MB-231 cells incubated with the free form of OG-PTX also showed OG uptake (**Figure 3**).

### *In vitro* Cytotoxicity of PTX-MSC-EMs Against MDA-MB-231 Cells

**FIGURE 4:**
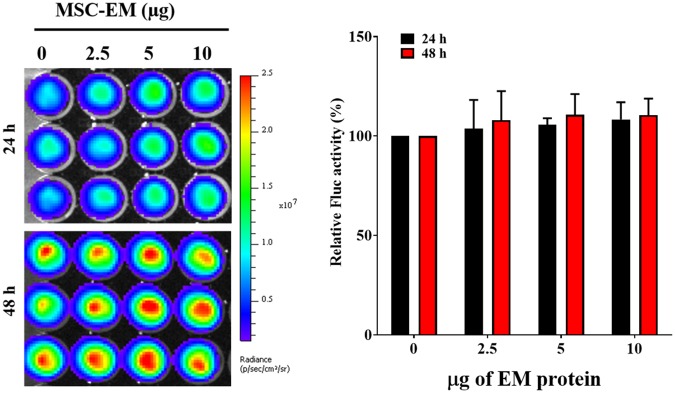
Fluc activity and quantitative evaluation of MDA-MB-231/effluc cells after treatment with MSC-EMs for 24 and 48 h. The values are expressed as the mean ± standard deviation (SD) of three experiments.

**FIGURE 5:**
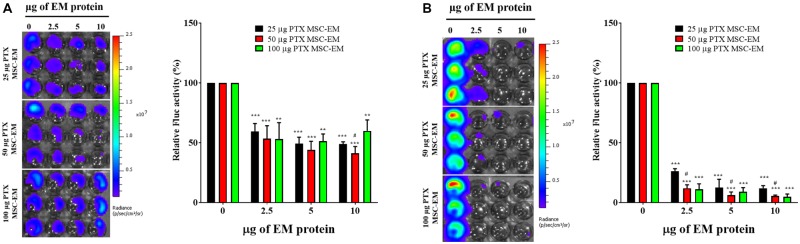
Fluc activity and quantitative evaluation of MDA-MB-231/effluc cells after PTX-MSC-EM treatment. **(A)** 24 h BLI and quantitative data. **(B)** 48 h BLI and quantitative data. Values are expressed as the mean ± standard deviation (SD). ^∗∗^*p* < 0.01 and ^∗∗∗^*p* < 0.001 (Student’s *t*-test). Significant difference was compared between individual control and treatment groups (25, 50, and 100 μg/mL of PTX-MSC-EM). ^#^*p* < 0.05 by Student’s *t*-test. (#) represents comparison between groups treated with 25 and 50 μg/mL of PTX-MSC-EMs.

No significant change was observed in the Fluc activity of MDA-MB-231/effluc cells following treatment with MSC-EMs (**Figure 4**). However, a decrease in the Fluc activity of MDA-MB-231/effluc cells was observed following treatment with PTX-MSC-EMs in a concentration-dependent manner (**Figure 5**). The decrease in the relative Fluc activity with 2.5, 5, and 10 μg of PTX-MSC-EM protein at 24 h was 59%, 49%, and 48% following treatment with 25 μg of PTX-loaded MSC-EMs; 53%, 44%, and 41% after treatment with 50 μg of PTX-loaded MSC-EMs; and 52%, 51%, and 59% after treatment with 100 μg of PTX-loaded MSC-EMs, respectively. At 48 h, the decrease in the relative Fluc activity was 26%, 12%, and 11% with 25 μg of PTX-MSC-EMs; 11%, 6%, and 5% with 50 μg of PTX-MSC-EMs; and 10%, 8%, and 4% with 100 μg of PTX-MSC-EMs, respectively. The cytotoxicity observed with 50 μg of PTX-loaded MSC-EMs was significantly higher (*p* < 0.05) than that observed with 25 μg of PTX-loaded MSC-EMs; no significant difference in cytotoxicity was observed between groups treated with 50 and 100 μg PTX-loaded MSC-EMs (**Figure 5**). Cell viability assessment with MTT assay (**Supplementary Figure S3**) showed a significant (*p* < 0.001 and *p* < 0.05) decrease in the viability of MDA-MB-231/effluc cells. We also performed the effect of MSC-EM to another breast cancer cell line MCF-7, found that no significant changes were observed after MSC-EM treatment for 24 and 48 h (**Supplementary Figure S4A**). Further the PTX-loaded MSC-EM to normal breast cells MCF-10A, we found that the PTX can kill the normal cells (**Supplementary Figure S4B**), as EVs can also deliver drugs to normal cells. If the EVs based targeted therapy is specific to cancer cells may not kill the nearby normal cells. Therefore, further studies need to test the targeted delivery of EM loaded with drugs to cancer cells.

### *In vivo* Therapeutic Effect of PTX-MSC-EMs Against Breast Cancer

**FIGURE 6:**
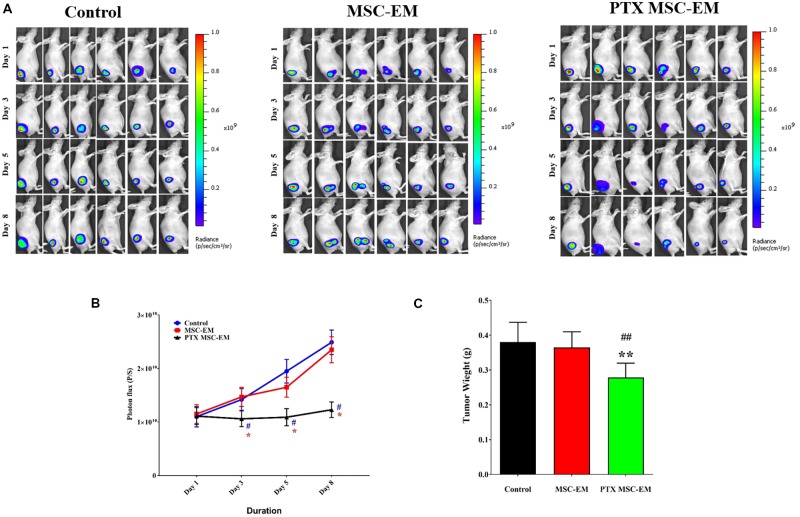
*In vivo* therapeutic effect of PTX-MSC-EMs on MDA-MB-231/effluc breast cancer xenograft. **(A)** Fluc activity of breast cancer cells (MDA-MB-231/effluc) measured by BLI in mice treated with either MSC-EMs or PTX-MSC-EMs. BLI images were taken on days 1, 3, 5, and 8 in six mice. **(B)** Quantitative Fluc activity data shown in A. **(C)** Tumor weights at the end of the study. BLI activity is shown in photon flux (photons/sec [p/s]). ^∗^*p* < 0.05 and ^∗∗^*p* < 0.01 for comparison between control and MSC-PTX-EM group. ^#^*p* < 0.05, ^##^*p* < 0.01 for comparison between MSC-EM and MSC-PTX-EM group.

The therapeutic effect of PTX-MSC-EMs was assessed in a xenograft MDA-MB-231 cancer mouse model. Xenograft mice carrying MDA-MB-231/effluc tumors were intratumorally injected with MSC-EMs or PTX-MSC-EMs on days 1 and 5. The Fluc activity of MDA-MB-231 xenograft was serially monitored using IVIS Lumina III imaging system. A significant (*p* < 0.05) inhibition in the tumor growth was observed in mice treated with PTX-MSC-EMs as compared to those treated with MSC-EMs and PBS (**Figures 6A,B**). At the end of the experiment, the tumors were excised and weighed. A significant reduction (*p* < 0.05) in tumor weights was observed for mice treated with PTX-MSC-EMs (**Figure 6C**).

## Discussion

Therapeutic strategies for improved delivery of drugs using engineered MSCs may be useful for managing cancers (Menon et al., 2009; Loebinger and Janes, 2010). MSCs isolated from different tissues such as the adipose tissue and bone marrow exhibit the capability to encapsulate and release anticancer drugs without any genetic changes, resulting in the inhibition of cancer growth both *in vitro* and *in vivo* (Pessina et al., 2011). Stem cells isolated from various sources are better candidates for regenerative medicine (Gorodetsky and Schäfer, 2011) and have possible oncologic applications for the inhibition of tumor progression in some tumor models (Khakoo et al., 2006; Qiao et al., 2008; Cousin et al., 2009). MSC-derived EVs offer several advantages over MSCs and have potential applications as drug delivery vehicles (Kim and Kim, 2017). For EV-mediated therapy, MSCs are the most important donor cell types, owing to their production efficiency, immune-regulating capacity, and clinical applicability (Yeo et al., 2013). EVs derived from stem cells are non-immunogenic and hence may not be easily cleared from the systemic circulation, thereby increasing the drug delivery efficiency to target tissues (Johnsen et al., 2014; Wiklander et al., 2015). However, mammalian cells release minimum amounts of exosomes (Lakhal and Wood, 2011; van Dommelen et al., 2012). The successful use of exosomes as drug delivery vehicles necessitates its large-scale production. Therefore, the alternative separation of exosome-related vesicles that have significantly higher yields is suitable for the development of nanosized DDSs. In the current study, human MSC-derived EMs and PTX-loaded EMs were obtained and serially extruded with polycarbonate membranes of varying pore sizes (10, 5, and 1 μm) in the presence and absence of various concentrations of PTX. The morphology and size of EM and the presence of EV markers such as CD63 and ALIX were confirmed and the absence of GM130 and calnexin were demonstrated, as shown in the previous studies (Gangadaran et al., 2017a; Kim et al., 2018). Furthermore, the production of MSC-EMs was more than eight-fold higher with more than 26-fold higher total protein as compared to the production of natural MSC-exosomes. A similar trend has been reported in previous studies and Exo and EMs reported to have same morphology (Hwang et al., 2015; Lunavat et al., 2016) but it is important to note that crucial components (proteins, lipids, nucleic acids) of exosomes are still largely unknown, and the integrative studies are required to elucidate the biological functions of these components.

One of the important challenges is the efficient loading of exosomes without causing significant changes to the structure and size of exosomal membranes. We purified PTX-MSC-EMs with various concentrations of PTX (25, 50, and 100 μg/mL). We found that the loading efficiency was 38.9, 76.1, and 74.22 ng/μg of EM proteins with 25, 50, and 100 μg/mL concentrations of PTX, respectively. A previous study on nanovesicle-based therapy with doxorubicin had shown increased entrapment of doxorubicin into nanovesicles through an increase in the concentration of doxorubicin (Jang et al., 2013). The size of EMs increased after drug loading, but no statistically significant difference was observed. Moreover, no change in the spherical shape of EM was observed following drug loading. Thus, the loading of PTX failed to have any significant effect on EM, suggestive of the suitability of this method. Furthermore, the amount of PTX loaded inside EM increased after increasing the concentration of PTX from 25 to 50 μg/mL; no further increase in the loading efficiency was observed at PTX concentrations of 50-100 μg/mL. After PTX loading, the presence of PTX in EMs and its delivery to cancer cells were confirmed by experiments using OG-PTX. These results are consistent with the studies, wherein microvesicles loaded with PTX were internalized into cells and accumulated in the vesicles instead of microtubules (Pessina et al., 2011; Pascucci et al., 2014).

Klopp et al. suggested that microvesicles and exosomes released from MSCs may exhibit a specific mechanism to transport their components into the microenvironment of the tumor to regulate tumor progression. Microvesicles isolated from MSCs have shown to significantly inhibit cancer cell proliferation (Bruno et al., 2012; Kalimuthu et al., 2016). In the current study, isolated MSC-EMs had no influence on the activity of Fluc in MDA-MB-231 cells (**Figure 3**). However, PTX-loaded MSC-EMs induced death in MDA-MB-231 cells, and the cytotoxicity was increased by increasing PTX concentration, as verified with bioluminescence imaging and MTT assay. These results demonstrate that MSC-EMs may carry and deliver chemotherapeutics to cancer cells. We treated normal breast cell line MCF-10A with PTX-MSC-EM. Although not extensively as cancer cells, drug (PTX) loaded MSC-EM also killed the normal cells. Therefore it is needed to study the targeted delivery of EM based drugs to breast cancer in living subjects.

To determine the *in vivo* antitumor activity of PTX-MSC-EMs, we established an MDA-MB-231/effluc breast cancer xenograft model with BALB/c nude mice. After tumor development, 50 μg of MSC-EMs and PTX-MSC-EMs were intratumorally injected twice during the treatment period. Bioluminescence imaging at day 8 revealed the significant inhibition in MDA-MB-231/effluc tumor growth. *Ex vivo* tumor weight was lower in mice receiving PTX-MSC-EMs than those from other groups. Jang et al. showed that doxorubicin-loaded vesicles isolated from Raw264.7 cells increased the apoptosis of cells surrounding the tumor and reduced the proliferation of colon cancer cells (Jang et al., 2013). Results of the current study and those reported by Jang et al. suggest that drug-loaded EMs may be one of the promising therapeutic armors for cancers. In the current study, we successfully loaded PTX in MSC-EMs using a simple procedure and demonstrated its anticancer effects both *in vitro* and *in vivo*. MSC-EMs may be one of the novel and promising alternatives as targeted drug delivery vesicles. Further studies are warranted to develop specific tumor-targeting MSC-EMs for successful clinical translation.

## Conclusion

MSC-EMs were successfully isolated using a simple procedure and drug-loaded MSC-EMs revealed therapeutic efficiency against breast cancer both *in vitro* and *in vivo*. MSC-EMs may be used as drug delivery vehicles for breast cancer treatment.

## Author Contributions

SK conceived, designed, and performed the experiments, as well as analyzed and interpreted the data, and drafted the manuscript. B-CA contributed to the study by conceiving and designing experiments, revising the manuscript, and approving the final version. PG, RR, LZ, JO, HL, AG, and SB performed the experiments. SJ, S-WL, and JL contributed reagents, materials, analysis tools, and revised the manuscript. All authors have approved the final version of the manuscript.

## Conflict of Interest Statement

The authors declare that the research was conducted in the absence of any commercial or financial relationships that could be construed as a potential conflict of interest.

## Supplementary Material

FIGURE S1 Phenotype marker analysis of MSCs. Analysis of CD44 and CD45 expression in MSCs by flowcytometry.

FIGURE S2 BLI of effluc activity and quantitative analysis of stably transduced MDA-MB-231/effluc cells with increased cell number. Data are expressed as the mean ± standard deviation (SD) of triplicate experiments.

FIGURE S3 Viability of MDA-MB-231/effluc cells after MSC-EM and PTX-MSC-EM treatment as analyzed by MTT assay. **(A)** Relative cell viability after MSC-EM treatment for 24 and 48 h. **(B)** Relative cell viability after PTX-MSC-EM treatment for 24 and 48 h. Values obtained from three experiments are expressed as the mean ± standard deviation (SD). ^∗∗∗^*p* < 0.001 (by Student’s *t*-test). The significant difference between individual control group versus treatment group (25, 50, and 100 μg/mL of PTX-MSC-EM). ^#^*p* < 0.05 for comparison between groups treated with 25 and 50 μg/mL PTX-MSC-EMs.

FIGURE S4 Effect of MSC-EM and PTX-MSC-EM on MCF-7 and MCF-10A cells. **(A)** Relative cell viability of MCF-7 breast cancer cells after MSC-EM treatment for 24 and 48 h. **(B)** Relative cell viability of normal breast cells (MCF-10A) after PTX-MSC-EM treatment for 24 and 48 h. Values obtained from three experiments are expressed as the mean ± standard deviation (SD). ^∗^*p* < 0.05; ^∗∗∗^*p* < 0.001 (by Student’s *t*-test).
