# Supplementary figures and images for: A New Approach for Loading Anticancer Drugs Into Mesenchymal Stem Cell-Derived Exosome Mimetics for Cancer Therapy
Source: Front Pharmacol. 2018 Sep 26;9:1116. doi: 10.3389/fphar.2018.01116 (PMC6168623; doi:10.3389/fphar.2018.01116)

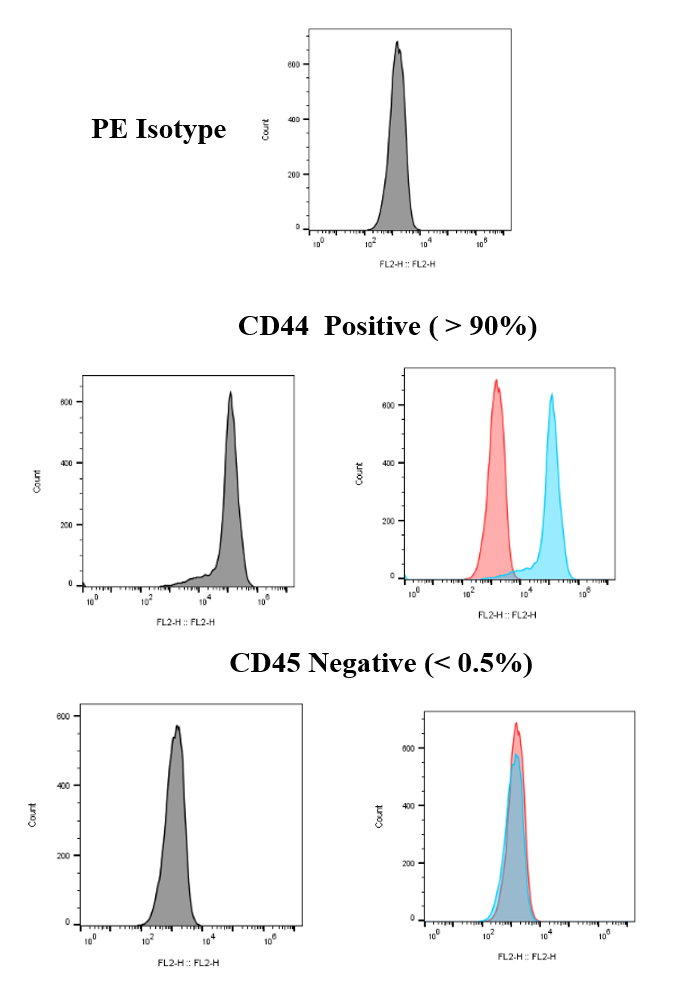

Supplement: FIGURE S1 — Phenotype marker analysis of MSCs. Analysis of CD44 and CD45 expression in MSCs by flowcytometry. [file Image_1.TIF]

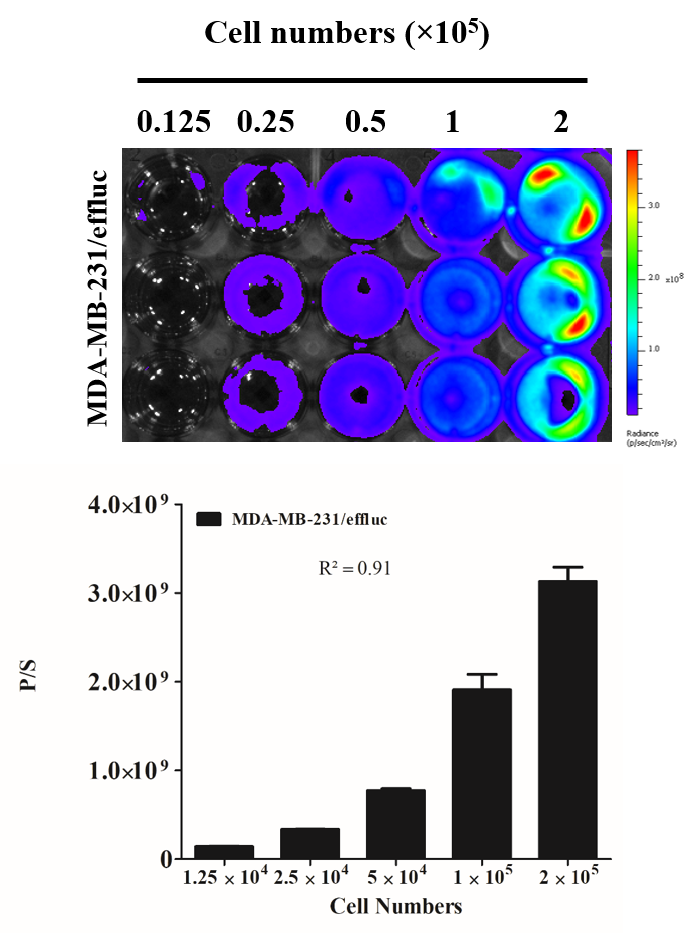

Supplement: FIGURE S2 — BLI of effluc activity and quantitative analysis of stably transduced MDA-MB-231/effluc cells with increased cell number. Data are expressed as the mean ± standard deviation (SD) of triplicate experiments. [file Image_2.TIF]

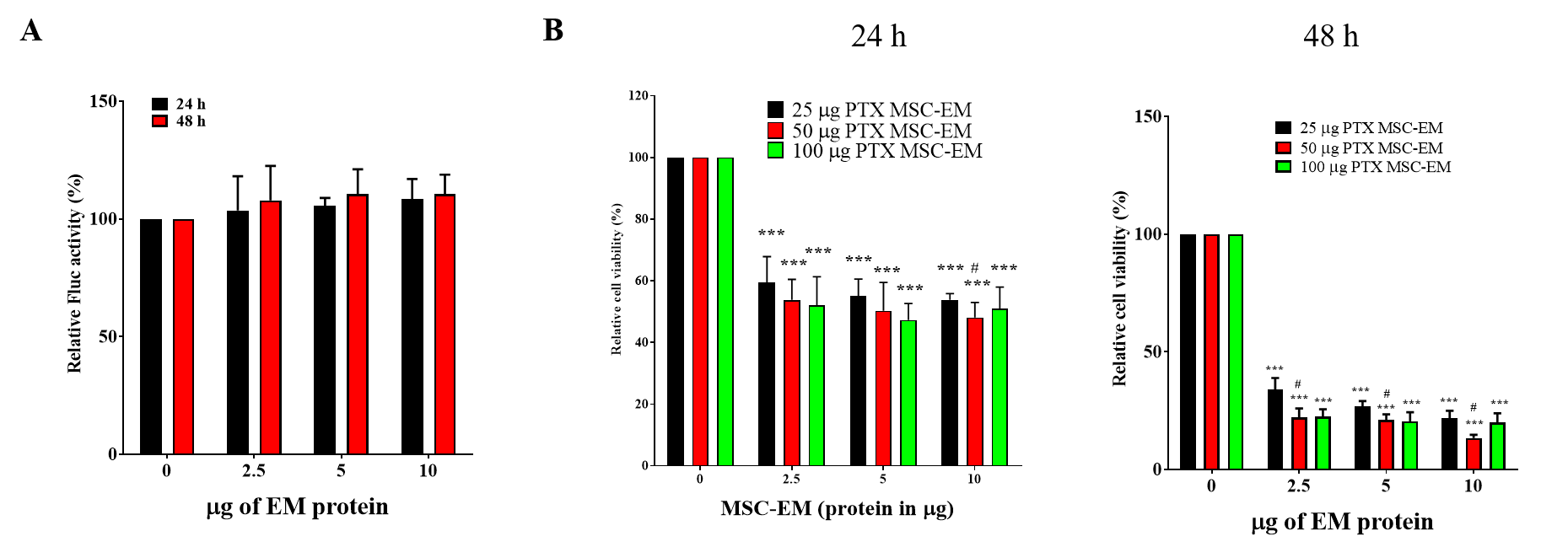

Supplement: FIGURE S3 — Viability of MDA-MB-231/effluc cells after MSC-EM and PTX-MSC-EM treatment as analyzed by MTT assay. (A) Relative cell viability after MSC-EM treatment for 24 and 48 h. (B) Relative cell viability after PTX-MSC-EM treatment for 24 and 48 h. Values obtained from three experiments are expressed as the mean ± standard deviation (SD). ∗∗∗p < 0.001 (by Student’s t-test). The significant difference between individual control group versus treatment group (25, 50, and 100 μg/mL of PTX-MSC-EM). #p < 0.05 for comparison between groups treated with 25 and 50 μg/mL PTX-MSC-EMs. [file Image_3.TIF]

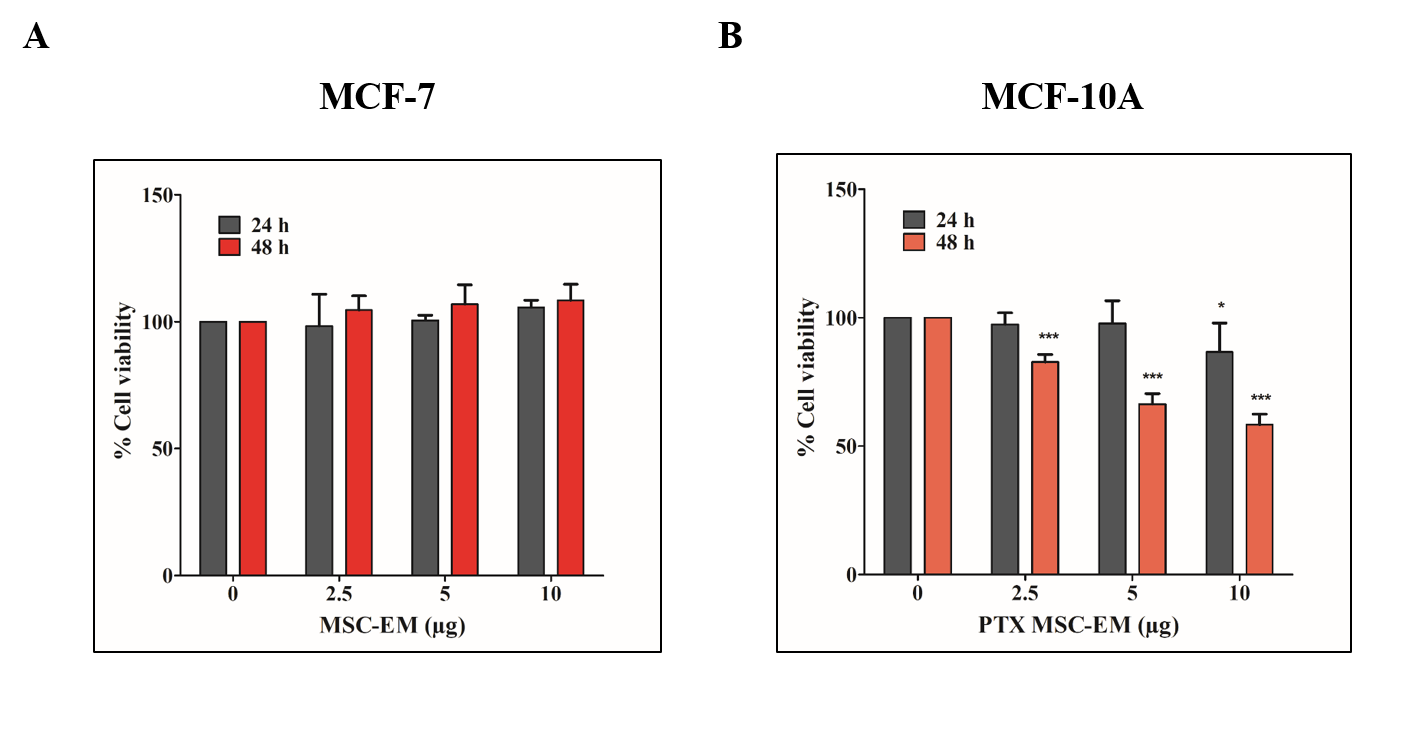

Supplement: FIGURE S4 — Effect of MSC-EM and PTX-MSC-EM on MCF-7 and MCF-10A cells. (A) Relative cell viability of MCF-7 breast cancer cells after MSC-EM treatment for 24 and 48 h. (B) Relative cell viability of normal breast cells (MCF-10A) after PTX-MSC-EM treatment for 24 and 48 h. Values obtained from three experiments are expressed as the mean ± standard deviation (SD). ∗p < 0.05; ∗∗∗p < 0.001 (by Student’s t-test). [file Image_4.TIF]
